# Activation of septal OXTr neurons induces anxiety- but not depressive-like behaviors

**DOI:** 10.1038/s41380-021-01283-y

**Published:** 2021-09-06

**Authors:** Tuanjie Huang, Fangxia Guan, Julio Licinio, Ma-Li Wong, Yunlei Yang

**Affiliations:** 1 Department of Medicine Division of Endocrinology, Albert Einstein College of Medicine, Bronx, NY USA; 2 School of Life Sciences, Zhengzhou University, Zhengzhou, Henan China; 3 Departments of Psychiatry and Neuroscience, SUNY Upstate Medical University, Syracuse, NY USA; 4 Department of Neuroscience, Albert Einstein College of Medicine, Bronx, NY USA; 5 Einstein-Mount Sinai Diabetes Research Center, Albert Einstein College of Medicine, Bronx, NY USA; 6 The Fleischer Institute for Diabetes and Metabolism, Albert Einstein College of Medicine, Bronx, NY USA

**Keywords:** Neuroscience, Depression

## Abstract

The neuropeptide oxytocin (OXT) is well recognized for eliciting anxiolytic effects and promoting social reward. However, emerging evidence shows that OXT increases aversive events. These seemingly inconsistent results may be attributable to the broad OXT receptor (OXTr) expression in the central nervous system. This study selectively activated septal neurons expressing OXTr using chemogenetics. We found that chemogenetic activation of septal OXTr neurons induced anxiety- but not depressive-like behavior. In addition, septal OXTr neurons projected dense fibers to the horizontal diagonal band of Broca (HDB), and selective stimulation of those HDB projections also elicited anxiety-like behaviors. We also found that septal OXTr neurons express the vesicular GABA transporter (vGAT) protein and optogenetic stimulation of septal OXTr projections to the HDB inactivated HDB neurons. Our data collectively reveal that septal OXTr neurons increase anxiety by projecting inhibitory GABAergic inputs to the HDB.

## Introduction

It has been well-demonstrated that anxiety is under the control of multiple neuronal populations and complex neural circuits primarily concentrated in the limbic system. Previous studies with local OXTr (oxytocin receptor) mRNA assays [1, 2] or OXTr reporter mice [3–5] have demonstrated that OXTr is widely expressed in mammalian brains, including the septum, nucleus accumbens, and ventral tegmental area [6, 7]. Previous studies have shown that the genes encoding the neuropeptide OXT are predominantly expressed in the magnocellular neurons of the paraventricular nucleus of the hypothalamus (PVN) and supraoptic nuclei (SON) [8]. In addition to the peripheral release of the processed OXT peptide from the posterior pituitary in response to various stimuli, oxytocinergic neurons in the PVN project to many different brain regions in the central nervous system (CNS) [9].

Previous studies have been focused on the functional roles of OXT in social reward-associated brain regions, including the nucleus accumbens [10] and ventral tegmental area [11, 12]. There is ample evidence showing that OXT elicits anxiolytic effects [13–17] and ameliorates depression [18], while emerging evidence indicates that OXT also exerts anxiogenic effects [19]. These seemingly inconsistent findings could be reconciled because the OXTr is widely expressed in the CNS, and different experimental paradigms were used in those studies. Neuronal populations participating in the anxiogenic effects of OXT remain to be identified.

Recently we showed that selective stimulation of glutamatergic inputs of the ventral hippocampus (vHPC) in the septum and septal vGAT (Vesicular GABA transporter) and vGluT2 (Vesicular glutamate transporter 2) neurons suppressed food intake [20–22], which suggests that the vHPC modulates hypothalamic feeding centers via the septum. We have also shown that feeding may change anxiety levels [23].

We observed that septal OXTr neurons expressed vGAT; however, their roles in regulating anxiety remain unclear. We have performed a series of experiments to define septal OXTr neurons’ roles in regulating anxiety- and depressive-like behavior and deciphered their downstream target. We employed a chemogenetic DREADD (Designer Receptor Exclusively Activated by Designer Drug) approach to manipulate cell-type-specific neuronal populations in a spatiotemporal manner. Our results show that DREADD stimulation of septal OXTr neurons or their projections in the HDB (Horizontal diagonal band of Broca) increases anxiety- but not depressive-like behaviors. We also found that activation of septal OXTr neuron projections to the HDB inhibits HDB neurons. Together, previous literature and our results in this study let us posit that septal neurons modulate anxiety- and depressive-like behaviors by projecting to different downstream targets.

## Methods

The Albert Einstein College of Medicine’s Institutional Animal Care and Use Committees approved all experimental protocols, which were conducted according to the US National Institutes of Health guidelines for animal research.

### Animals

We used both male and female C57BL/6 J and *Oxtr*-Cre (Jax 031303, Jackson Lab, Bar Harbor, ME) mice (age 5–8 weeks) at the start of experiments. We group-housed 3–5 mice per cage in temperature- (22–25 ^o^C) and humidity-controlled rooms on a 12-h light:12-h dark cycle, with lights on from 8:00 am to 8:00 pm and with ad libitum access to mouse standard chow (PicoLab Rodent Diet 20, 5058, LabDiet, St. Loius, MO) and water. Mice were single-caged after they received viral transductions with or without guide cannula or fiber insertions until we finished all the experimental procedures. The virally transduced mice were randomly and evenly matched for age and sex.

### Pharmacology

All chemicals were purchased from Sigma (Millipore Sigma, St Louis, MO) except for JHU37160 (J60; water-soluble) which was purchased from HelloBio Inc (Princeton, NJ). We performed intraperitoneal (i.p.) injections using 27-gauge needles and diluted the stocks of J60 in saline (200 μl) at the final dose of J60 (1 mg/kg) on the experimental days. For experiments requiring intra-HDB administration, an injector (33 GA, Plastics One) with a 1-mm extension beyond the guide cannula (3.5 mm length; 26 GA, Plastics One) was attached to a Hamilton syringe via polyethylene tubing. We used a micromanipulator (Narishige International Inc, Amityville, NY) to control the injection speed at 50 nl per min for 2 min and withdrawn the injector 2 min after the final injection. The guide cannula was inserted into the brain for intra-HDB injections and anchored to the skull using grip cement (Dentsply Sirona, Charlotte, NC). A dummy cannula (33 GA, Plastics One, Torrington, CT) was used to prevent cannula clogging. The amount for intra-HDB was 100 nl of J60 (1 mg /kg).

### Viral Vectors

Viral vectors used in this study included: AAV vectors for the Cre-dependent hM3Dq (AAV_2_-hSyn-DIO-hM3Dq-mCherry, addgene#44361, titer at 7.8 × 10^12^ vg/ml), hM4Di (AAV_2_-hSyn-DIO-hM4Di-mCherry, addgene#44362, titer at 1.27 × 10^13^ vg/ml), or ChR2 [(AAV5-EF1a-DIO-hChR2(H134R)-EYFP-WPRE-HGHpA), addgene#20298, titer at 1.0 × 10^13^ vg/ml]. AAV vectors for non-Cre dependent GCaMP_6s_ (AAV_1_-hSyn1-GCaMP6s-P2A-nls-dTomato, addgene#51084, titer at 5.0 × 10^12^ vg/ml; AAV_1_-hSyn1-axon-GCaMP6s, addgene#111262, titer at 1.0 × 10^13^ vg/ml). Control vectors were used (AAV-hSyn-DIO-mCherry, addgene#50459, titer at 1.5 × 10^13^ vg/ml; AAV-hSyn-EGFP, addgene#50465, titer at 8.0 × 10^12^ vg/ml). Aliquoted viral vectors were stored at −80 °C until used for stereotaxic injections.

### Stereotaxic viral injections and cannula implantation for intra-HDB injections

Surgical procedures have been detailed in our recent publications [20, 21, 24]. Briefly, mice were anesthetized with isoflurane (1.5–3.0%) and placed in a stereotaxic frame (Harvard Apparatus, Holliston, MA). The mouse skull was exposed via a small incision, and two small holes were drilled directly above the viral injection sites bilaterally using a micro-precision drill (CellPoint Scientific, Gaithersburg, MD). A glass pipette with 20–40 µm tip diameter was inserted into each side of the brain, and two injections (300 nl each side) of the vectors were delivered into the lateral septum at coordinates (bregma +0.5 mm; midline ±0.4 mm; dorsal surface −2.2 mm), or into the vHPC at coordinates (bregma −3.2 mm; midline +3.2 mm; dorsal surface −3.8, −3.5, −3.2 mm). A micromanipulator (Narishige) was used to control the viral injection at a speed of 30 nl per min, and the injection pipette was withdrawn 15 min after the final injection to assure adequate viral delivery. For the experiments using intra-HDB injections, a customer-made bilateral 26-gauge stainless steel cannula (3.5 mm length, Plastics One) was inserted through the craniotomy. For photometry experiments, a TeleFipho fiber-optic cannula (fiber core at 400 μm / NA 0.39, cladding at 425 μm, ferrule diameter at 2.5 mm; Amuza Inc) was implanted over the vHPC (coordinate at bregma −3.2 mm; midline +3.2 mm; dorsal surface −3.0 mm) or over the lateral septum (coordinate at bregma +0.5 mm; midline +0.4 mm; dorsal surface −2.0 mm). The guide cannula or fiber-optic cannula was anchored to the skull with grip cement (Dentsply). Stainless dummies inserted into the guide cannula prevented the guide cannula from clogging. Optic fiber dust caps were placed on optic fibers to keep the fibers clean. Mice were returned to their home cages and singly housed typically for at least two weeks for recovery and viral expression before performing the experiments.

### Open field test (OFT)

OFTs were performed following the procedures detailed in our previous studies [23, 25, 26]. In brief, virally transduced mice were habituated to the behavioral room for 2 h before beginning the experimental sessions. The open field consisted of a brightly lit 40 × 40 cm arena. We defined the center of the arena at 20 × 20 cm area. Mice received i.p. J60 (1 mg/kg) 30 min before the 10-min test using ANY-maze software (Stoelting Co., Wood Dale, IL). Between the trials, we cleaned the arena with 70% ethanol.

### Elevated-plus maze (EPM)

Similar to the OFT, virally transduced mice were habituated to the behavioral room for 2 h. We placed the animals in the center of the start zone and allowed them to explore the arms for 5 min, 30 min after receiving i.p. J60 (1 mg/kg). ANY-Maze software (Stoelting Co.) monitored and recorded the animals’ exploration. The arena was cleaned with 70% ethanol between trials.

### Sucrose preference test (SPT)

We followed the procedure as described in previous studies [27] with modifications. Briefly, we adapted the mice in their home cages for 72 h to two liquid diet feeding tubes (50 ml; Bio-SerV, #9019, Flemington, NJ) containing 1% sucrose in water (wt/vol) and tap water, respectively. We changed the positions of the two tubes every 24 h. Mice had free access to lab chow. Each animal was given one sucrose tube and one water tube for 14 h (7:00 pm to 9:00 am) during the baseline measurement. During the preference test on the following day, mice were deprived of both food and water for 12 h (7:00 am to 7:00 pm). After which and 30 min before the test, mice were treated with J60 or vehicle via i.p. injections and provided with one sucrose tube and one water tube for 14 h (7:00 pm to 9:00 am). The sucrose solution and water weights were recorded, and the percent sucrose preference index was calculated using the following formula: Sucrose preference index = [sucrose consumption/ (water + sucrose consumption)] × 100%.

### Social behavioral test

Thirty min after i.p. J60 or vehicle treatment, two age- and sex-matched mice were placed in one chamber (20 × 30 cm). As described in previous studies [28, 29], exploratory behaviors were recorded for 20 min and analyzed using a social behavior software (Video Capturing and TopScan High-Throughput Option Version 3.0, CleverSys Inc., Reston, VA). Between the trials, the arena was cleaned with 70% ethanol. Active contact, passive contact, social approach, social leave, social follow, and social sniff were recorded. Bouts of social interaction and social interaction duration were calculated for every group using the formula: social interaction = social follow + social sniff.

### Electrophysiology and optogenetic stimulation

Following our recently published protocols [20, 22, 24], acute coronal sections that included the septum and/or HDB were prepared from the septal OXTr neuron ChR2-, hM3Dq-, hM4Di-, or control fluorescent protein-transduced *Oxtr-*Cre mice. Briefly, mice were deeply anesthetized with isoflurane and decapitated. Mouse brains were dissected rapidly and placed in ice-cold oxygenated (95% O_2_ and 5% CO_2_) solution containing (in mM): 110 Choline Chloride, 2.5 KCl, 1.25 NaH_2_PO_4_, 2 CaCl_2_, 7 MgSO_4_, 25 D-glucose, 3.1 Na-pyruvate and 11.6 Na-L-ascorbate, pH 7.3. Coronal brain slices (260-μm thickness) were cut with a vibratome (Leica; VT 1200 S) and maintained in an incubation chamber at 34 ^o^C for 30 min, and then brought to room temperature until being transferred to a recording chamber. During experiments, an individual slice was transferred to a submersion-recording chamber and continuously perfused with recording solution containing the following (in mM): 119 NaCl, 25 NaHCO_3_, 11 D-glucose, 2.5 KCl, 1.25 MgCl_2_, 2 CaCl_2_, and 1.25 NaH_2_PO_4_, aerated with 95% O_2_/5% CO_2_ (1–2 ml per min at 30 ^o^C). Virally transduced neurons or their projections were identified using an Olympus microscope (BX51WI) and observed by fluorescence emission. Loose-seal cell-attached recordings were made through the experiments using an Axo-patch 700 B amplifier (pClamp 11.0 software, Molecular Devices, San Jose, CA). The recording electrodes had tip resistances of 3–5 MΩ and seal resistances of 20–70 MΩ. Recording pipettes were filled with artificial cerebrospinal fluid (aCSF). Recordings were initiated 5 min after the loose-seal cell-attached recordings were established. For the DREADD experiments, J60 was added to the circulating aCSF after 2–5 min of baseline recordings. For the ChR2 experiments, blue lights were delivered on the surface of brain slices using an optic fiber connected to a blue laser power (Crystal Laser 473 nm), and light pulse (3 ms duration) was controlled by the pClampfit software. Responses were digitized at 10 kHz through whole experiments using Multi-Clamp 700B amplifier and analyzed with pClampfit 11 software (Molecular Devices). For detection of action potentials, we used template matching (pClampfit) followed by visual inspection.

### Wireless fiber photometry (TeleFipho) in freely moving mice

For experiments performing TeleFipho recordings, vHPC neurons or their axons were transduced with GCaMP_6s_. A unilateral TeleFipho fiber-optic cannula (400 μm core, 0.39 numeric aperture, 425 μm cladding, and 2.5 mm diameter; Amuza Inc) was implanted over the vHPC or the lateral septum. A TeleFipho system (Amuza Inc), including the small and light-weighted headstage and wireless transmission circuit, was set and adjusted with technical help. The headstage (3 g weight; 12 × 12 x 22 mm dimensions) is composed of a filter cub, photodetector, and blue light source (excitation wavelength peaked at 470 nm with 445–490 nm filter band; emission wavelength with a 500–550 nm band). The sampling rate was set at 100 Hz with a gain at 10^10^ V/A. The signal transmission distance was within 2 m. The receiver containing a 1x photometry analog out and 1x general-purpose analog were connected to a PC installed with the TeleFipho software. The light power was adjusted to reach a baseline of around 35000 following the software instructions to achieve maximum sensitivity and avoid saturating the detector. The maximum power of the 465 nm was 300 µW. We normalized the GCaMP_6s_ signal intensity (I) for each trial and calculated the Z score as (I − I_mean_)/I_SD_, where I_mean_ and I_SD_ represent the mean and standard deviation of the signals for each mouse. The TeleFipho experiments were performed 5 min after connecting the optic fibers to the animals and before placing the mice in the open arms of the EPM or the light chamber of a light-dark box (LDB), respectively, for 5- or 10-min free explorations. The LDB consists of a box (60 cm × 40 cm x 20 cm) divided into a small (20 cm × 40 cm x 20 cm) dark chamber and a large (40 cm×40 cm x 20 cm) brightly lit chamber. A small opening (5 cm high by 4 cm wide) located at floor level in the center of the dividing plate connects the two chambers.

### Immunofluorescence assays

Mice were perfused with 0.1 M phosphate-buffered saline (PBS) in pH 7.4, followed by 4% paraformaldhyde (PFA) in PBS and then 0.1 M PBS. Septal coronal brain sections (40 μm) were obtained using a vibratome (Leica VT1200, Leica Biosystems, Buffalo Grove, IL) and mounted on glass slides. The virally transduced mice received i.p. injections of J60 30 min before the perfusion. Briefly, brain sections were washed three times in washing buffer (0.1% Triton X-100 in 0.1 M PBS) for 10 min, followed by permeabilization in a solution containing 1% Triton X-100 in 0.1 M PBS) for 30 min at room temperature, and then blocked for 2 h in a blocking solution (5% bovine serum in washing buffer). Brain sections were then incubated overnight at 4 °C with primary antibodies diluted in PBS, supplemented with 1% BSA and 0.1% Triton X-100, and washed three times and incubated overnight at 4 °C with Alexa fluoro 647-conjugated anti-Fos antibody (E-8) (1:150; sc-166940; Lot#A3020; Santa Cruz Biotechnology Inc., Dallas, TX); and the vGAT was stained using vGAT Polyclonal antibody (1:25, Invitrogen, Thermo Fisher Scientific, Waltham, MA) respectively. Sections were washed three times using washing buffer for 2 h at room temperature, incubated with the goat anti-rabbit IG-conjugated with Alexa fluoro 594 (1:1,000; Invitrogen) for vGAT-stain at room temperature for 2 h, and then washed three times for 10 min each time using washing buffer and mounted on glass slides with Fluoromount-G mounting medium (Southern Biotech, Birmigham, AL). Images (2 µm thickness, five images) were collected using 20X and 40X objectives in a confocal microscope (Leica SP8).

### Statistics and reproducibility

Animals were randomly assigned to experimental (DREADD) or control groups (control fluorescent protein transduced) before viral injections and behavioral experiments. Only mice with accurate viral injections and cannula placements were included in the data analysis. All experiments were repeated twice for each mouse, and average values were calculated for each mouse for statistical analysis. Differences between two groups were analyzed using Student’s *t*-tests or Mann-Whitney tests when appropriate. Comparison of more than two groups was made using one-way ANOVA with Tukey’s post hoc test. Repeated measures (RM) two-way ANOVA with the within-subject factors of time segment and treatment (vehicle vs. J60) or mixed ANOVA with the within-subject factor of time segment and the between-subjects factor of viral injections type was used to analyze data from more than two groups across various time points. Sidak’s post hoc test was used to test from significant effects at various time segments following the detection of a significant main effect or interaction. Homoscedasticity and normality parameters were obtained in male and female data. All data were analyzed using Prism 8.0 (GraphPad Software).

## Results

### DREADD activation of OXTr expressing neurons in the septum

**Fig. 1:**
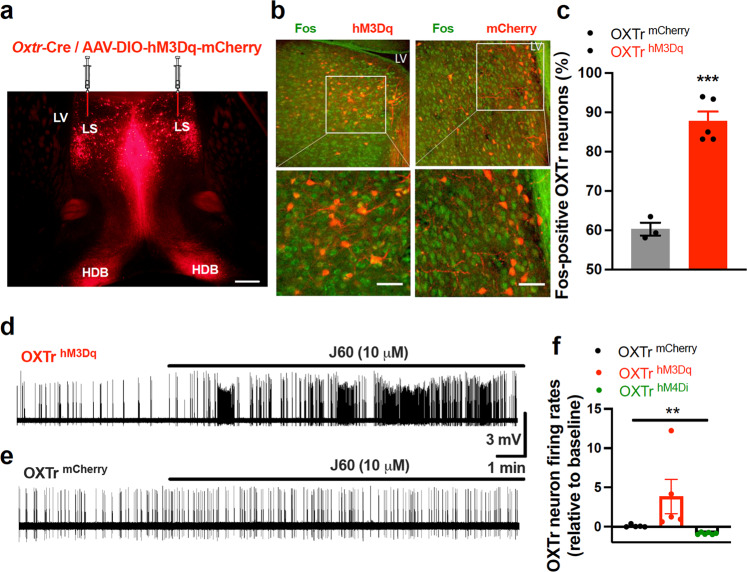
DREADD activation of septal OXTr neurons. **a** A representative image of hM3Dq-mCherry-transduced OXTr neurons in the septum and their projections to the HDB. **b**, **c** J60 treatment via i.p. injections potently increased Fos positive OXTr neurons in the hM3Dq-mCherry transduced mice (*n* = 5) compared to mCherry-transduced mice (*n* = 3): (**b**) Representative images of Fos and hM3Dq-mCherry in the septal OXTr neurons of hM3Dq-mCherry transduced animals; (**c**) Quantification of the percentage of Fos positive OXTr neurons transduced with hM3Dq-mCherry (hM3Dq, *n* = 5; mCherry; *n* = 3; *p* = 0.0002; Unpaired student *t* test). **d**–**f** Loose-seal cell-attached recordings: adding J60 to the circulating aCSF increased the firing rates at the (**d**) hM3Dq-transduced OXTr neurons but not the (**e**) mCherry-transduced neurons in acute brain slices of the virally transduced *Oxtr*-Cre mice; (**f**) Group data of action potential firing rates relative to baseline in hM3Dq- hM4Di-, or mCherry-transduced OXTr neurons with J60 (*n* = 5 per group). One-way ANOVA was used for (**f**), and an unpaired student *t*-test for (**c**). Mean ± SEM; **p < 0.01; ***p < 0.001. Scale bars, 2 mm for (**a**); and 50 µm for (**b**).

We first validated septal OXTr neuron activation with a chemogenetic DREADD approach, which was employed to selectively stimulate or inhibit these neurons in *Oxtr*-Cre transgenic mice. Following our previously published protocols [20, 21, 24], we targeted vectors respectively carrying Cre-dependent stimulatory hM3Dq, inhibitory hM4Di, or control mCherry to the lateral septum (LS) in *Oxtr-*Cre mice (Fig.1a), and the proteins were predominantly expressed in the septum and OXTr neuron projections to downstream targets including the HDB (Fig. 1a). We applied the in vivo DREADD agonist JHU37160 dihydrochloride (J60), which has been verified in our recent studies [24], to evaluate the ability of J60 to regulate OXTr neuron activity. We observed that i.p. injection of J60 (1 mg/kg) significantly increased Fos-positive OXTr neurons in the hM3Dq-transduced mice compared to the mCherry-transduced ones (Fig. 1 b, c).

Moreover, we performed in vitro loose-seal cell-attached recordings from septal OXTr neurons in acute brain slices in the virally transduced *Oxtr*-Cre mice. J60 (10 μM) was added to the circulating aCSF after a stable baseline recording. Action potential firing rates were analyzed and compared before and after the application of J60. Consistent with the above Fos results, we observed that J60 potently increased the action potential firing rates in the hM3Dq-transduced OXTr neurons compared to mCherry-transduced neurons (Fig. 1d–f). In addition, J60 decreased the action potential firing rates of hM4Di-transduced OXTr neurons (Fig. 1f). These results indicate that the DREADD approach with J60 treatment can be reliably utilized to correlate neuron activities to animal behaviors.

### DREADD activation of septal OXTr neurons induces anxiety

**Fig. 2:**
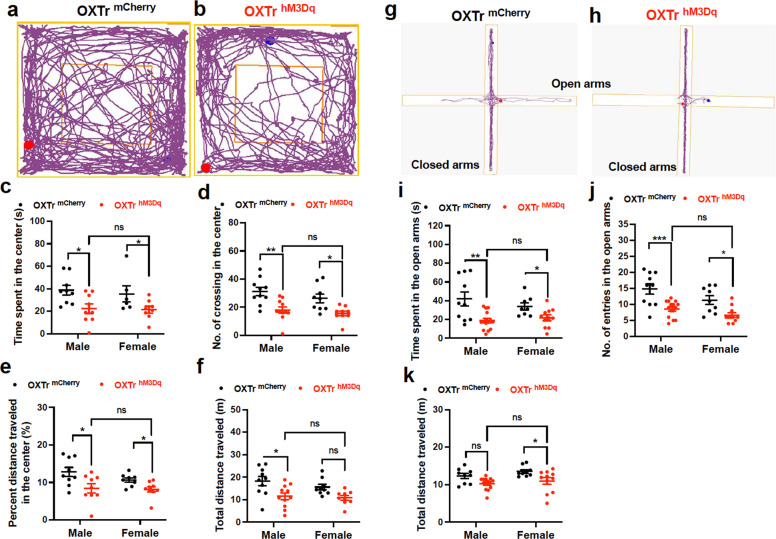
Activation of septal OXTr neurons induced anxiety in both males and females. **a**, **b** Representative open field test (OFT) tracks of the septal OXTr neuron of (**a**) mCherry- or (**b**) hM3Dq-transduced Oxtr-Cre mice. **c** Time spent in center of the open field (male mCherry, *n* = 9; male hM3Dq, *n* = 9; female mCherry, *n* = 6; female hM3Dq, *n* = 8). **d** The number of entries in the center of the open field. **e** Percent distance traveled in the center. **f** Total traveled distance during the OFT. **g**, **h** Representative elevated plus-maze (EPM) tracks of the septal OXTr neuron of (**g**) mCherry- or (**h**) hM3Dq-transduced mice (male mCherry, *n* = 10; male hM3Dq, *n* = 15; female mCherry, *n* = 9; female hM3Dq, *n* = 11. **i** Time spent in the open arms of the EPM. **j** The number of entries in the open arms. **k** Total distance traveled in the EPM. Mice were treated with J60 i.p. 30 min before the OFT and EPM tests. Two-way ANOVA was used to analyze all panels. Student t-tests were used for female data (**c**, **i**). Mean ± SEM; *p < 0.05; **p < 0.01; ***p < 0.001; ns not significant.

We performed a series of animal behavioral tests to evaluate the septal OXTr neurons’ capability to regulate anxiety- and depressive-like behaviors by activating or inactivating septal OXTr neurons of hM3Dq-, hM4Di-, or mCherry-transduced mice respectively. The virally transduced mice received J60 treatment via i.p. injections 30 min before the behavioral tests. We observed that DREADD activation of septal OXTr neurons induced anxiety-like behaviors in both males and females, as indicated by the decreased time spent and number of entries in the center of the open field (Fig. 2a–d), and decreased percent center distance (Fig. 2e) in activated hM3Dq-transduced mice compared to the mCherry-transduced mice. We also noticed that septal OXTr neuron activation in male mice significantly decreased total distance in the OFT (Fig. 2f).

**Fig. 3:**
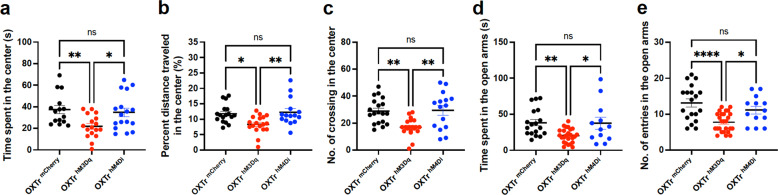
Inactivation of septal OXTr neurons did not elicit anxiety behavior. **a**–**c** Open-field behavioral tests (OFTs) were performed in the septal OXTr neuron in hM3D (*n* = 23)-, hM4Di (*n* = 17)-, or mCherry (*n* = 20)-transduced mice: (**a**) Time spent in the center of open field; (**b**) Percent distance traveled in the center; and (**c**) The number of entries in the center of the open field. **d**, **e** Elevated plus-maze (EPM) tests were performed in the septal OXTr neuron of hM3Dq (*n* = 26)-, hM4Di (*n* = 13)-, or mCherry (*n* = 19)-transduced mice: (**d**) Time spent in the open arms of the EPM and (**e**) The number of entries in the open arms. Mice were treated with J60 i.p. 30 min before the OFT and EPM tests. One-way ANOVA was used to analyze all panels. Mean ± SEM; *p < 0.05; **p < 0.01; ***p < 0.001; ****p < 0.0001; ns not significant.

To further confirm the anxiogenic effects of septal OXTr neurons, we performed the EPM test. Consistent with the OFT findings, we observed that activation of the septal OXTr neurons with J60 via i.p. injections diminished the time spent (Fig. 2g–i) and the number of entries in the EPM’s open arms (Fig. 2j) in hM3Dq-transduced mice compared to mCherry-transduced animals, and hM3Dq-transduced female mice had a small but significant decreased in the total distance traveled (Fig. 2k). DREADD inactivation of septal OXTr neurons with i.p. injection of J60 did not exert effects in hM4Di-transduced mice compared to mCherry-transduced mice, as indicated by no differences in the time spent in the center, center distance, and the number of crossing in the center of the open field (Fig. 3a–c), and no differences in the time spent in open arms and the number of entering the open arms (Fig. 3d, e) of the EPM.

### Activation of septal OXTr neuronal projections to HDB induces anxiety-related behaviors

**Fig. 4:**
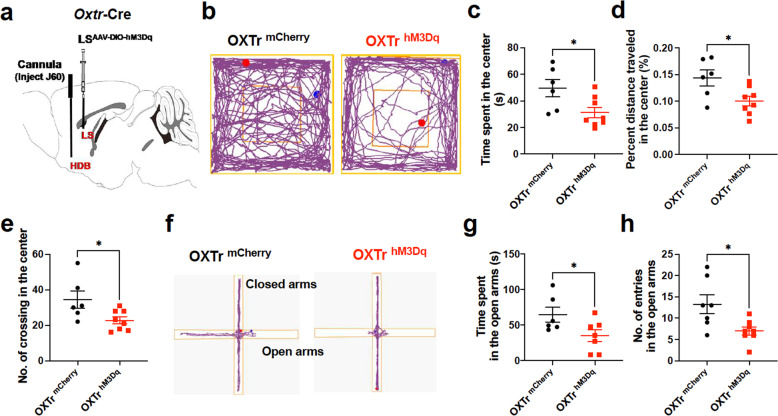
Selective stimulation of septal OXTr neuron projections to HDB induced anxiety. **a** Schematic illustration of experimental design. A vector carrying Cre-dependent hM3Dq or mCherry was bilaterally targeted to the lateral septum, and a guide cannula was implanted for intra-HDB injections in Oxtr-Cre mice. **b** Representative tracks of the open field test (OFT) in the septum of OXTr hM3Dq (right, *n* = 8)- and mCherry (left, *n* = 6)-transduced mice. **c** Time spent in the center of the open field. **d** Percent center distance traveled in the OFT. **e** The number of center entries in the OFT. **f** Representative traces of the EPM recorded in the septal OXTr hM3Dq (right, *n* = 8)- and mCherry (left, *n* = 7)-transduced mice. **g** Time spent in the open arms of the EPM. **h** The number of entries in the open arms. Two-tailed unpaired Student’s *t*-tests were used to analyze all panels. Mean ± SEM; *p < 0.05; ns not significant.

We next tried to identify the circuitry underlying the anxiogenic effects of septal OXTR neurons. As stated above, we observed that septal OXTr neurons project dense fibers to the HDB (Fig. 1a). To determine the behavioral relevance of septal OXTr neural projections to the HDB, we transduced septal OXTr neurons with the stimulatory hM3Dq by injecting a vector carrying Cre-dependent hM3Dq in the lateral septum of *Oxtr*-Cre mice and implanting a guide cannula to perform intra-HDB injections (Fig. 4a). We observed that activating septal OXTr neural projections with intra-HDB J60 injections induced anxiety, as indicated by the decreased time spent in the center (Fig. 4b–c), percent distance traveled in the center (Fig. 4d), and number of center entries (Fig. 4e) of the open field in the OXTr neuron hM3Dq-transduced mice compared to the OXTr neuron mCherry-transduced mice. To further confirm these projections’ anxiogenic effects, we performed the EPM test (Fig. 4f–h). Matching with the above OFT results, we observed that stimulation of OXTr neural projections by J60 via intra-HDB injection decreased the time spent in the open arms (Fig. 4f, g) and open arm entering number (Fig. 4h) in the OXTr neuron hM3Dq-transduced mice compared to the mCherry-transduced mice.

### Septal OXTr neurons do not modulate social and sucrose preference behaviors

**Fig. 5:**
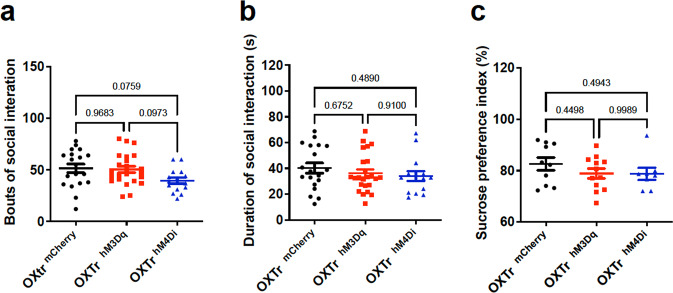
Septal OXTr neurons had minimal effects on social interactions and depression-like behavior. No significant differences were detected in the septal OXTr neuron of hM3Dq (*n* = 24)-, hM4Di (*n* = 15)-, and mCherry (*n* = 19)-transduced mice in social behavior and anhedonia tests. **a**–**b** Social interaction test. **c** Sucrose preference test. Mean ± SEM.

To evaluate whether septal OXTr neurons modulate animal sociability, we performed social behavioral tests by placing one pair of virally transduced age- and gender-matched mice in one open chamber for 20 min, following published protocols [28, 29]. Time engaged in social interactions, including the social following and head-body-genital sniffing for the paired mice (combining behaviors for both mice), were recorded and analyzed using TopScan software (CleverSys Inc.). We observed that chemogenetic activation of septal OXTr neurons with J60 via i.p. injections did not elicit effects on social behaviors, as there were no differences in the bouts (Fig. 5a) and duration (Fig. 5b) of social interactions between the septal OXTr neuron hM3Dq-, hM4Di-, and mCherry-transduced mice. We also performed the sucrose preference test (SPT) in the transduced animals and noticed that i.p. injection of J60 did not cause alterations in sucrose preference among them (Fig. 5c). These results indicate that septal OXTr neurons do not modulate sociability and sucrose preference, suggesting that these neurons do not regulate depressive-like behaviors.

### Optogenetic stimulation of septal OXTr projections to the HDB inhibits HDB neurons

**Fig. 6:**
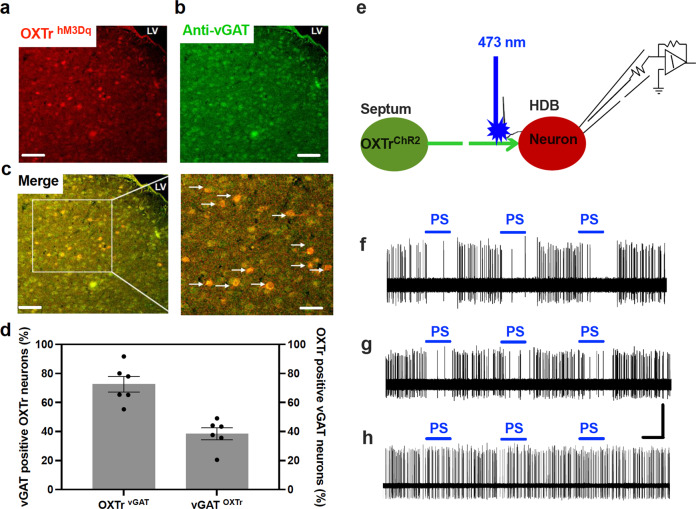
Optogenetic stimulation of septal OXTr projections to the HDB inactivated HDB neurons. **a**–**c** Representative images of (**a**) hM3Dq-mCherry-transduced septal OXTr neurons, (**b**) anti-vGAT stains, and (**c**) overlap of hM3Dq-mCherry and vGAT. **d** Quantification of the percentage of vGAT-expressing OXTr and OXTr-expressing vGAT neurons (*n* = 6 per group). **e** A schematic illustration of loose-seal cell-attached recordings at HDB neurons with optogenetic stimulation of ChR2-expressing OXTr projections to the HDB in acute brain slices of the OXTr neuron from ChR2-transduced *Oxtr*-Cre mice. **f**–**h** Optogenetic stimulation of septal OXTr neuron projections to the HDB decreased the action potential firing rates at eight of the 15 recorded HDB neurons: (**f**, **g**) two representative traces of PS-induced decrease in action potential firing rates recorded at two HDB neurons, and (**h**) one representative trace that PS did not affect the firing rates. Mean ± SEM. Scale bars, 50 µm for (**a**, **b**, and **c**); 1 s and 1 mV for f, g, and h. PS photostimulation at 20 Hz.

Previous studies demonstrate that the lateral septum is mainly composed of GABAergic neurons [30, 31], and the vGAT is a neuronal marker of GABAergic neurons in the central nervous system [32, 33]. We thus posited that septal OXTr neurons are a subpopulation of septum vGAT neurons. Accordingly, we stained septal OXTr neurons using anti-vGAT antibodies in brain sections of *Oxtr*-Cre mice transduced with hM3Dq-mCherry in the septal OXTr neurons. Consistently, we observed that most virally transduced OXTr neurons expressed vGAT, and approximately forty percent of vGAT-positive neurons expressed OXTr (Fig. 6a–d).

To further define the inhibitory projections of septal OXTr neurons to the HDB, we evaluated the HDB neuron action potential firing rates with optogenetic stimulation of OXTr neuron projections to the HDB in ChR2-transduced *Oxtr*-Cre mice (Fig. 6e). We performed loose-seal cell-attached recordings on HDB neurons in acute brain slices. Consistently, we observed that optogenetic stimulation of the ChR2-positive OXTr neuron projections to the HDB by shining blue light on the surface of the slices decreased the action potential firing rates in 8 of 15 recorded HDB neurons (Fig. 6f–h).

### The vHPC and its projections to the septum are activated under anxiety

**Fig. 7:**
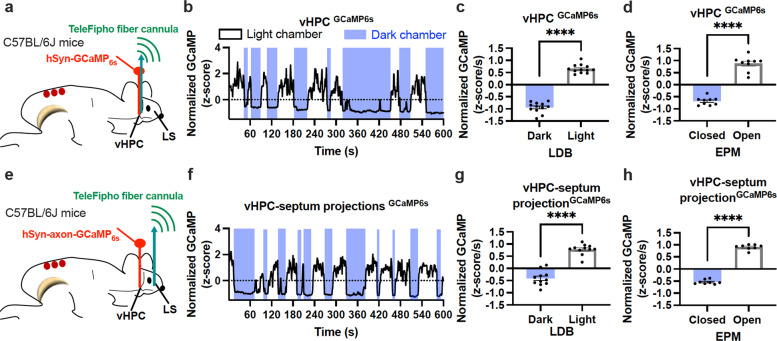
The vHPC and its projections to the septum were excited under anxiogenic environments. **a** A schematic illustration of targeting a vector carrying GCaMP_6s_ to the vHPC and implanting a wireless TeleFipho fiber cannula over the targeted site. **b** A representative trace of real-time wireless photometry monitoring of vHPC GCaMP_6s_ signals in light and dark chambers of the light-dark box (LDB). **c**, **d** Group data of normalized GCaMP_6s_ signals recorded in (**c**) LDB and (**d**) elevated plus-maze (EPM) tests. **e** An illustration of targeting a vector carrying axon-GCaMP_6s_ to the vHPC and implanting a wireless photometry fiber over the LS. **f** A representative trace of real-time monitoring of vHPC axon GCaMP_6s_ signals in the LS in the LDB. **g**, **h** Group data of normalized vHPC axon GCaMP_6s_ signals in the LS recorded in (**g**) LDB and (**h**) EPM. Each dot in (**c**, **d**, **g**, **h**) represents one animal. Mean ± SEM; ****p < 0.0001; vHPC ventral hippocampus, LS lateral septum.

It remains unclear about the stimulatory drive to the septum under anxiogenic conditions. Our recent study shows that the vHPC [22], a well-established emotional brain region, project excitatory inputs to the septum. To define the activity of the vHPC under anxiety, we performed real-time wireless photometry monitoring of vHPC neuron activity in freely moving mice. We targeted a vector carrying the Ca^2+^ indicator GCaMP_6s_ to the vHPC to transduce hippocampal neurons with the protein of GCaMP_6s_ and implant a TeleFipho fiber-optic cannula over the virally targeted brain region (Fig. 7a).

After two weeks that allowed for GCaMP_6s_ expression and one week of acclimation to carrying a headstage 15 min per day, mice were habituated to the behavioral test room 30 min before placing them in the light chamber of the LDB for 10 min with simultaneous real-time photometry monitoring of GCaMP_6s_ signals. We observed that the average intensity of GCaMP_6s_ signals recorded in the light chamber was more robust than in the dark chamber (Fig. 7b, c). Matching with the LDB results, we also evaluated vHPC neurons with the EPM, and GCaMP_6s_ signals were increased when mice were exploring in the open arms compared to the closed arms (Fig. 7d). In cohort groups, we evaluated hippocampal projections to the septum. We transduced vHPC neurons with a vector carrying an axon-targeted Ca^2+^ indicator GCaMP_6s_ and implanted a TeleFipho cannula over the lateral septum (Fig. 7e). Consistently, we observed that the intensity of GCaMP_6s_ signals recorded on hippocampal projections to the lateral septum was increased when mice were in the light chamber of the LDB (Fig. 7f, g) or the open arms of the EPM (Fig. 7h).

## Discussion

It is well-recognized that OXT elicits anxiolytic effects [34, 35] and promotes social reward [10–17] in rodents; however, human studies show that OXT administration increases recall of aversive events or stressful stimuli [19, 36, 37]. Furthermore, overexpression of OXTr in the septum exacerbates stress-induced fear [38]. These seemingly inconsistent findings might be attributable to different experimental paradigms and reconciled because OXTr is widely expressed in different brain regions. The functional roles of septal OXT signaling in anxiety and mood regulation remain to be defined, and the involved downstream targets await identification.

The septum nucleus is a limbic brain structure implicated in various cognitive and emotional processes [30, 39, 40]. Our recent studies show that chemo/optogenetic stimulation of ventral hippocampal glutamatergic inputs in the septum suppressed food intake [22], and activated septal vGAT and vGluT2 neurons reduced food intake by projecting to the lateral hypothalamus and paraventricular nucleus, respectively [20, 21]. These results suggest that hippocampal suppression of feeding probably modulate hypothalamic feeding centers via the septum. This study observed that septal neurons expressing OXTr also expressed vGAT; however, it remains unclear whether and how these neurons participate in anxiety regulations. In this study, we found that chemogenetic activation of septal OXTr neurons induced anxiety-related behaviors. HDB probably mediated this anxiogenic effect as chemogenetic stimulation of septal OXTr neuron projections to the HDB elicited similar anxiogenic effects.

Meanwhile, optogenetic stimulation of the septal OXTr projections to the HDB inactivated the neurons localized within the HDB in our electrophysiology experiments. These results let us posit that septal OXTr neurons exerted anxiogenic effects probably by inhibiting HDB neurons via inhibitory GABAergic projections to the HDB. Therefore, our results suggest that the HDB neurons participated in the septal OXTr neuron anxiogenic effects; however, we cannot exclude other neural circuit pathways as septal neurons project to different brain regions.

The diagonal band of Broca (DB) is a basal forebrain region that consists of vertical (VDB) and HDB [41] limbs, projecting to the perilimbic, infralimbic, medial prefrontal cortices [42], hypothalamus [43], central nucleus of the amygdala and the septal nuclei [44]. Meanwhile, different neuronal populations, including cholinergic, GABAergic, and glutamatergic neurons, are intermingled in the HDB, and these neuronal populations project to different regions in mammalian brains [45–48]. It is also important to mention that approximately 75 percent of septal OXTr neurons express vGAT and optogenetic stimulation of septal OXTr neuron projections to the HDB inhibited about 53 percent of HDB neurons, suggesting that OXTr neurons project to subpopulations of HDB neurons. Together, this septal and HDB neurochemical and anatomical heterogeneity determines the complexity of the neural circuits involved in the septal regulation of anxiety- and depressive-like behaviors, which are needed to be addressed in future studies. For example, a combined Cre-loxp and Flip-FRT system would be helpful to manipulate septal neurons and HDB cholinergic, glutamatergic and GABAergic neurons selectively.

There is emerging evidence indicating that cholinergic neurons send dense projections to the amygdala [46], a well-known brain region involved in anxiety regulation [49, 50], and cholinergic neurons express the inhibitory GABA neurotransmitters [45, 51]. These results let us posit that septal OXTr neuron inhibition of GABA neurons in the HDB would induce anxiety by stimulating the amygdala via HDB disinhibition, which would participate in vHPC-dependent anxiety. However, we cannot exclude the involvement of other circuitries. Furthermore, our data also show that the vHPC and its projections to the septum are activated under anxiety, suggesting that the vHPC-projected neurons localized within the septum, including the OXTr neurons, and the subsequent descending septum to HDB to amygdala circuitries, probably partake in the vHPC-dependent anxiety, which awaits identification in the future.

It also is necessary and timely to point out that our data show that activation of septal OXTr neurons does not modulate depressive-like and prosocial behavior. However, it is well-recognized that OXT actions promote multiple aspects of socio-sexual behaviors and drug-seeking behavior. The complex OXT signaling in multiple mammalian brain regions and different experimental paradigms might explain these seemingly inconsistent results [1, 52]. For example, earlier studies on the role of OXT in emotions have relied on systemic administration or knockdown, lacking cell-type specificity and spatiotemporal resolution. Our study avoided these limitations as we combined chemogenetics and Cre-transgenic animals, allowing us to manipulate neuron activities in a spatiotemporal manner selectively. These differential findings suggest that the neural circuitry for the septal regulation of anxiety and mood might be distinct or partially overlapping. Meanwhile, we observed that inactivation of septal OXTr neurons elicited minimal effects on anxiety- and depressive-like behaviors, which may imply that these neurons are silent in baseline conditions.

Increased locomotion may be a confounding factor in behavioral tests such as the forced swim test (FST) [53–55]. However, studies using a drug or an animal model of depression support that changes in immobility in the FST may occur independently of changes in other behaviors [53, 56, 57]. In our studies, locomotor activity change was significant but small and unlikely to impact the other behavioral tests. Other factors may also influence behavioral tests, such as pre-exposure to tail suspension test (TST) or other stressors [58]. Thus, if a drug or procedure is shown to modulate anxiety and locomotion, these effects do not necessarily need to be related.

Collectively, we report functional roles of septal OXT signaling in the regulation of anxiety and identified the involved downstream target, which might serve as a target for therapeutic interventions in minimizing unintended side effects of OXT.
